# Elevated serum A20 is associated with severity of chronic hepatitis B and A20 inhibits NF-κB-mediated inflammatory response

**DOI:** 10.18632/oncotarget.17153

**Published:** 2017-04-17

**Authors:** Hanchen Xu, Lei Wang, Peiyong Zheng, Yang Liu, Chunlei Zhang, Kaiping Jiang, Haiyan Song, Guang Ji

**Affiliations:** ^1^ Institute of Digestive Diseases, Longhua Hospital, Shanghai University of Traditional Chinese Medicine, Shanghai 200032, China; ^2^ China-Canada Centre of Research for Digestive Diseases, Shanghai 200032, China; ^3^ Department of Hepatology, Foshan Hospital of Traditional Chinese Medicine, Foshan 528000, China

**Keywords:** A20, chronic hepatitis B, serological biomarker, NF-κB, inflammation

## Abstract

A20 is a powerful suppressor for inflammatory response. This study aims to determine A20 level in patients with chronic hepatitis B (CHB), and analyze its association with the disease severity. The role of A20 in inflammatory response was further investigated *in vivo* and *in vitro*. Our results showed significantly higher A20 in both serum and liver tissues in CHB patients than in health controls. Serum A20 level was positively correlated with ALT, AST and TNF-α. To induce hepatitis with inflammation and liver injury, mice were injected intraperitoneally with D-galactosamine (D-GalN), resulting in rapid increase of A20 in serum and liver tissues. Consistently, HepG2 and Huh-7 cells exposed to Lipopolysaccharide (LPS) or D-GalN were promoted to express A20. Moreover, overexpression or knockdown of A20 inhibited or increased TNF-α secretion separately. A20 significantly reduced pro-inflammatory cytokines expression and down-regulated phospho-IκBα and phospho-p65 in both cells. In conclusion, elevated A20 expression is involved in the severity of CHB, suggesting A20 to be a possible serological biomarker for the disease prognosis. Additionally, the inflammatory response is attenuated by A20 through inhibiting NF-κB activity, which partially contributes to the hepato-protective function of this molecule. Thus, up-regulating A20 might be a potential strategy for preventing the progress of CHB.

## INTRODUCTION

Hepatitis B virus (HBV) infection is a global public health problem, with more than 350 million chronic carriers and estimated 40 million people with chronic hepatitis B (CHB) worldwide [1]. The clinical manifestations of CHB range from acute hepatitis to various phases of chronic infection, including inactive carrier state, chronic hepatitis, cirrhosis and hepatocellular carcinoma (HCC) [2, 3]. Due to periodic activation of the host immune system against infected hepatocytes, CHB is characterized by its dynamic persistent virus replication and liver inflammation. Around 1 million people died of liver failure, liver cirrhosis or HCC progressed from CHB each year [2, 3]. During the disease progresses, inflammatory response plays a pivotal role [4, 5].

Recent studies have demonstrated that patients with CHB showed a remarkable increase of NF-κB activation in liver biopsies, which closely correlated with the progression of disease [6–8]. And during liver injury, NF-κB was identified as a critical pro-inflammatory mediator [9, 10]. Evidences have also suggested that deprivation of NF-κB-mediated pro-inflammatory effects could be a rational strategy in the treatment of CHB. The activated NF-κB could induce multiple negative regulators, which subsequently contribute to inhibit further activation of inflammatory signaling pathways, thereby, protect the host from excessive inflammatory responses.

A20 (also known as tumor necrosis factor α inducible protein 3, TNFAIP3), a protein with seven zinc fingers, is an E3 ubiquitin ligase and identified as a key regulator of fundamental biological processes, such as immunity, inflammation and apoptosis. Normally, it is only expressed in a few types of cells at a low basal level. But its transcription is rapidly up-regulated upon stimulation by tumor necrosis factor (TNF), interleukin (IL)-1 or LPS, which activated NF-κB in most cell types including hepatocytes [11, 12]. It is well-known that A20 as a powerful suppressor for inflammatory response through inhibiting NF-κB activation and NF-κB-mediated cytokine signaling pathway [13–17]. Studies have revealed part of the molecular mechanisms, whereby A20 deubiquitinase activity and ubiquitin binding, linear ubiquitination, and cellular kinases cooperate to regulate inflammation and cell death [18, 19]. A20 homozygous knockout mice are born cachectic, and die within 3–6 weeks of birth from systemic inflammation, predominantly in the liver [13]. A20 deficient hepatocytes display sustained activity of NF-κB signaling and gene expression upon TNF or LPS challenge. Hepatocyte-specific A20 deficiency sensitizes mice to develop spontaneous liver inflammation, demonstrating the essential role of A20 in maintaining liver physiological immune homeostasis [20]. It was recently reported that A20 mRNA expression in peripheral blood mononuclear cells from CHB patients was elevated and associated with dynamic progression of chronic HBV infection [21]. However, another study showed Hepatitis B virus X protein (HBx) suppressed A20 expression in L-O2 and HepG2 cell lines [22]. By far, the A20 expression in liver, in hepatocytes or its secreted level in serum of patients with CHB as well as its biological function remain unclear. Therefore, in this study, we measured A20 in the serum and liver tissues from CHB patients and healthy controls, as well as analyzed its association with the related clinical parameters. In addition, *in vivo* and *in vitro* experiments were also performed to study the anti-inflammatory role and signaling pathway of this molecule.

## RESULTS

### Elevated serum A20 level is associated with the severity of chronic hepatitis B

**Figure 1:**
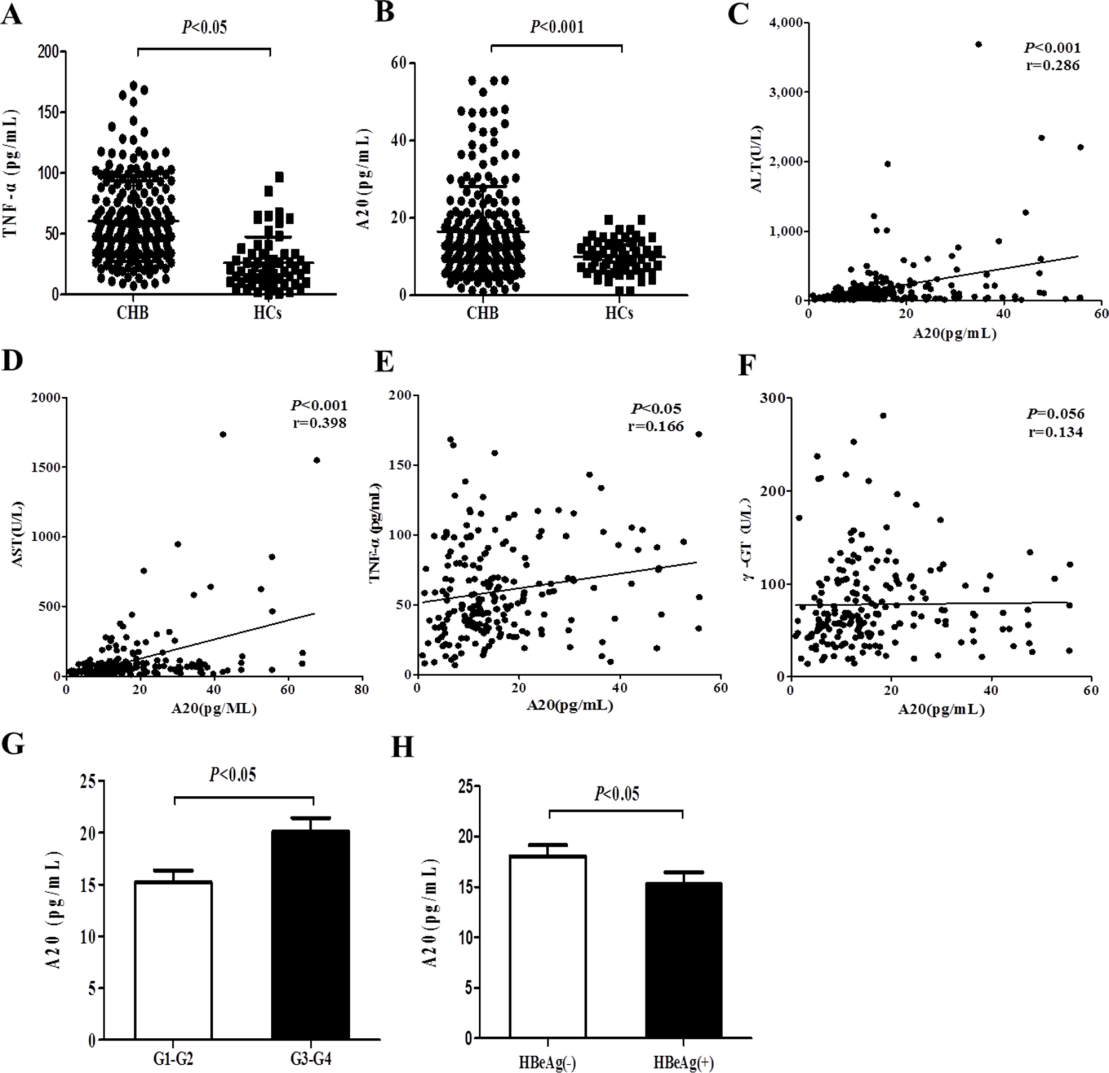
Elevated serum A20 level is associated with the severity of chronic hepatitis B (**A**–**B**) Serum A20 and TNF-α expression levels from patients with CHB (*n* = 205) and healthy controls (*n* = 60) were measured by ELISA. (**C**–**F**) Correlations between serum A20 and ALT, AST, γ-GT or TNF-α level were analyzed. (**G**) Comparison of serum A20 level in CHB patients with liver inflammation grade G3-G4 and G1-G2. (**H**) Comparison of serum A20 level in CHB patients with HBeAg (−) and HBeAg (+). Data are represented as mean ± SD.

The basic characteristics of all the enrolled participants including 205 patients with CHB and 60 healthy individuals are listed in Table 1. Liver damage exhibited by the elevated serum activity of ALT (*P* < 0.001), AST (*P* < 0.01), γ-GT in the patients with CHB compared with the healthy controls. As shown in Figure 1A, TNF-α expression level in serum was significantly increased in patients with CHB as compared to that in the controls. And serum A20 in patients with CHB was found significantly higher than that in HCs (*P* < 0.001) (Figure 1B).

**Table 1:** The basic characteristics of the enrolled participants

Variables	CHB (*n* = 205)	HCs (*n* = 60)	*P*-value
Male (%)	160 (78%)	46 (76.7)	
Age (years)	34 (16–62)	32 (18–51)	0.222
HBeAg (+) (%)	113 (55.1%)	NA	
ALT	195.5 (14.3–3686)	24 (18–36)	< 0.001
AST	110.6 (11.4–1736)	23 (15–36)	0.0012
γ-GT	78 (14.14–281.1)	27.82 (11–46)	< 0.001
HBV DNA+ (%)	183 (89.3%)	0	
G G1 (%)	26 (12.7%)	NA	
G2 (%)	63 (30.7)	NA	
G3 (%)	67 (32.7)	NA	
G4 (%)	49 (23.9)	NA	

CHB, chronic hepatitis B; HBeAg, hepatitis B e antigen; ALT, alanine aminotransferase; AST, aspartate aminotransferase; γ-GT, γ- glutamyltransferase; G, Grade of inflammatory activity; NA, not available.

To identify whether elevated A20 expression level in serum contributes to the severity of CHB, we evaluated the correlations between A20 expression level and clinical parameters in patients with CHB. The results demonstrated that level of A20 in serum was positively correlated with ALT (r = 0.286, *P* < 0.001), AST (r = 0.398, *P* < 0.001) and TNF-α (r = 0.166, *P* < 0.05) (Figure 1C–1E). There was no significant correlation between A20 and γ-GT (Figure 1F). CHB patients with the liver tissue inflammatory stage D3 and D4 had significantly higher serum A20 level than the stage G1 and G2 (Figure 1G). Additionally, serum A20 level was higher in CHB patients with HBeAg (−) compared to those with HBeAg (+) (Figure 1F). These results suggest that the elevated expression level of A20 in serum may be involved in the pathogenesis of CHB and closely associated with the degree of liver damage and inflammation of CHB.

### Increased expression of A20 in liver tissues from CHB patients

**Figure 2:**
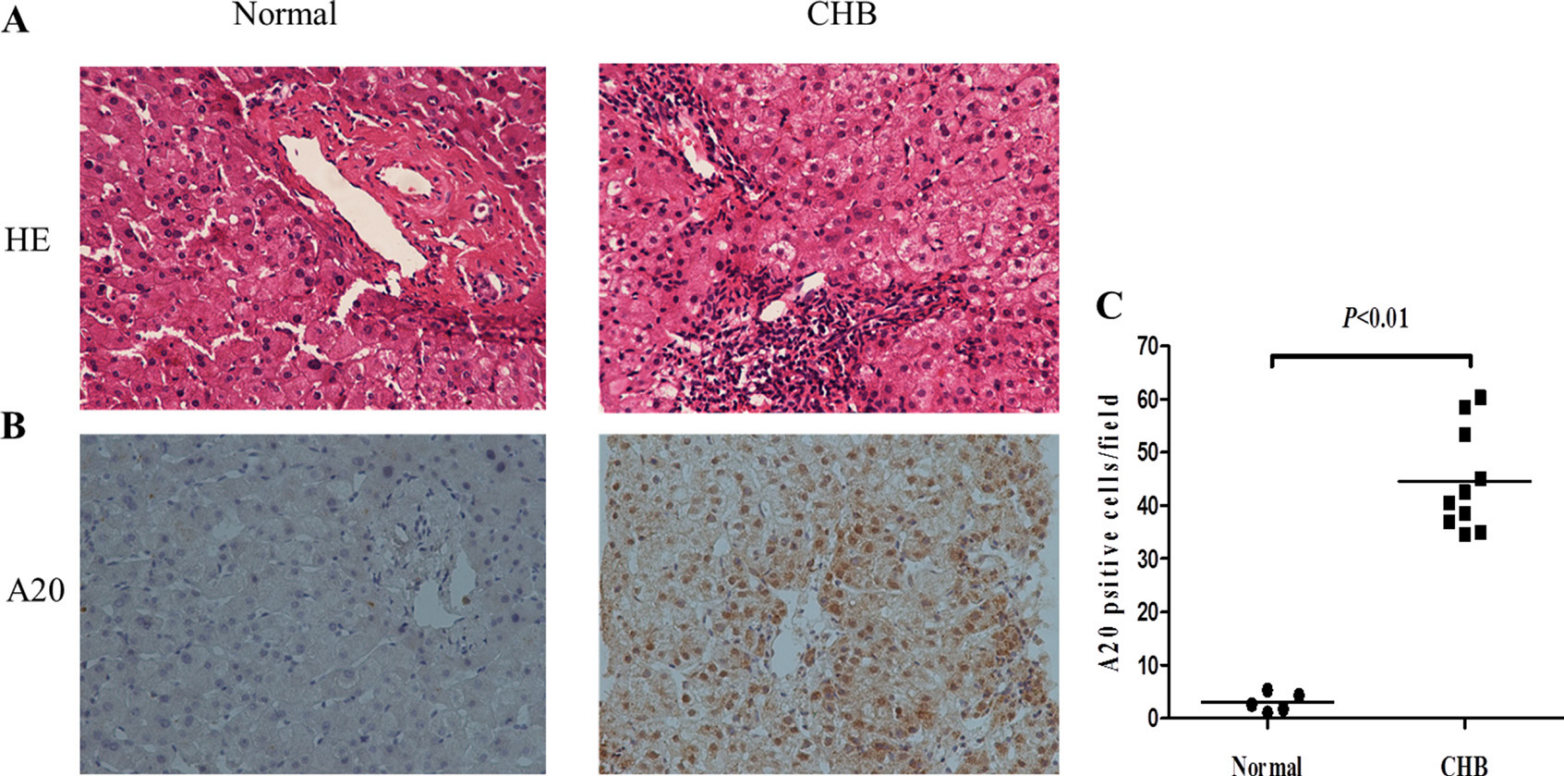
Increased expression of A20 in liver tissues from CHB patients (**A**) HE staining of liver sections from CHB patients and normal liver tissues. The original magnification is 200×. (**B**) Immunohistochemistry of liver biopsies from 10 CHB patients and 5 normal liver tissues with A20 antibody. The original magnification is 200×. (**C**) Quantification of A20-positive cells in each biopsy from patients with CHB compared with controls. Data are expressed as mean ± SD.

Liver sections stained with HE showed scattered inflammation and necrosis in liver tissues from CHB patients (Figure 2A). In order to know the origin of elevated serum A20, immunohistochemistry of liver biopsies from 10 CHB patients and 5 normal liver tissues as control was performed with antibody against A20. As Figure 2B–2C shows, increased positive stain of A20 in both hepatocytes and inflammatory cells was found in human liver tissues from CHB patients in comparison to normal controls (*P* < 0.01).

### Liver injury and A20 expression are induced by D-GalN in mice

**Figure 3:**
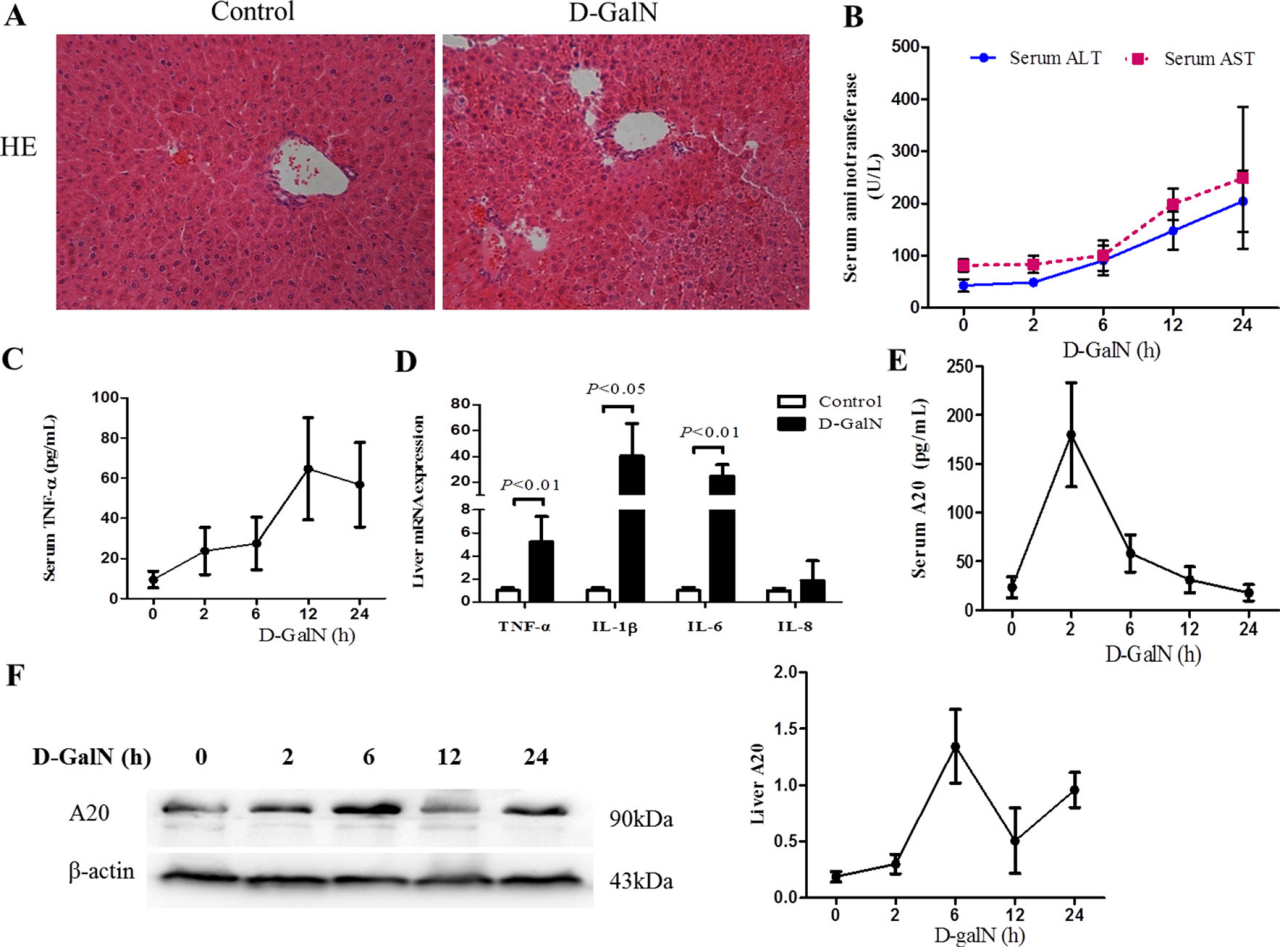
Liver injury and A20 expression are induced by D-GalN in mice (**A**) HE staining of liver sections from mice with liver injury induced by D-GalN or controls at 24 h after D-GalN administration. The original magnification is 200×. (**B**) Dynamic change of serum ALT and AST of mice after D-GalN injection (*n* = 10 per group). (**C**) Dynamic change of serum TNF-α of mice (*n* = 10 per group) (**D**) The mRNA levels of pro-inflammatory cytokines in mice liver tissues at 24 h after D-GalN administration were measured by real-time RT-PCR, with GAPDH as internal control (*n* = 4 per group). (**E**) Dynamic change of serum A20 levels in mice at different time point after D-GalN injection (*n* = 10 per group). (**F**) The kinetic hepatic A20 protein levels of mice were evaluated by immunoblot analysis. β-actin was determined as the loading control (*n* = 3 per group). Data are expressed as mean ± SD.

To investigate any potential correlation of A20 expression with inflammatory response during liver injury, we examined serum A20 and hepatic A20 expression in mice induced by D-GalN. HE staining of liver tissues showed scattered inflammatory cells infiltration and lightly changed lobule structure in the mice treated with D-GalN for 24 hours (Figure 3A). There was no significant change with serum ALT and AST at 2 h point after D-GalN injection, but subsequently their levels kept significantly increasing from 6 h to 24 h (Figure 3B), which suggested the development of hepatic injury had already begun. Serum TNF-α level increased constantly and significantly since 2 h after D-GalN administration, and reached the peak at 12 h (Figure 3C). We further examined the expression of pro-inflammatory cytokines in liver tissues by real-time PCR. Obviously elevated expression level of TNF-α, IL-1β and IL-6 was observed in mice liver collected at 24 h after D-GalN injection (Figure 3D). These results indicated liver inflammation in the mice induced by D-GalN.

Then we further detected the A20 expression in serum and liver tissues of the mice. Figure 3E shows that serum A20 level rapidly increased at 2 h after injecting D-GalN, but subsequently decreased gradually from 2 h to 24 h. Immunoblot analysis of dynamic hepatic A20 expression in the model mice showed that A20 expression was induced to increase obviously at 6 h, followed by a decrease at 12 h but then increase at 24 h again (Figure 3F). These dynamic changes of suggested A20 might be part of the response during hepatic injury and inflammation.

### A20 expression is induced by LPS and D-GalN in hepatocytes

**Figure 4:**
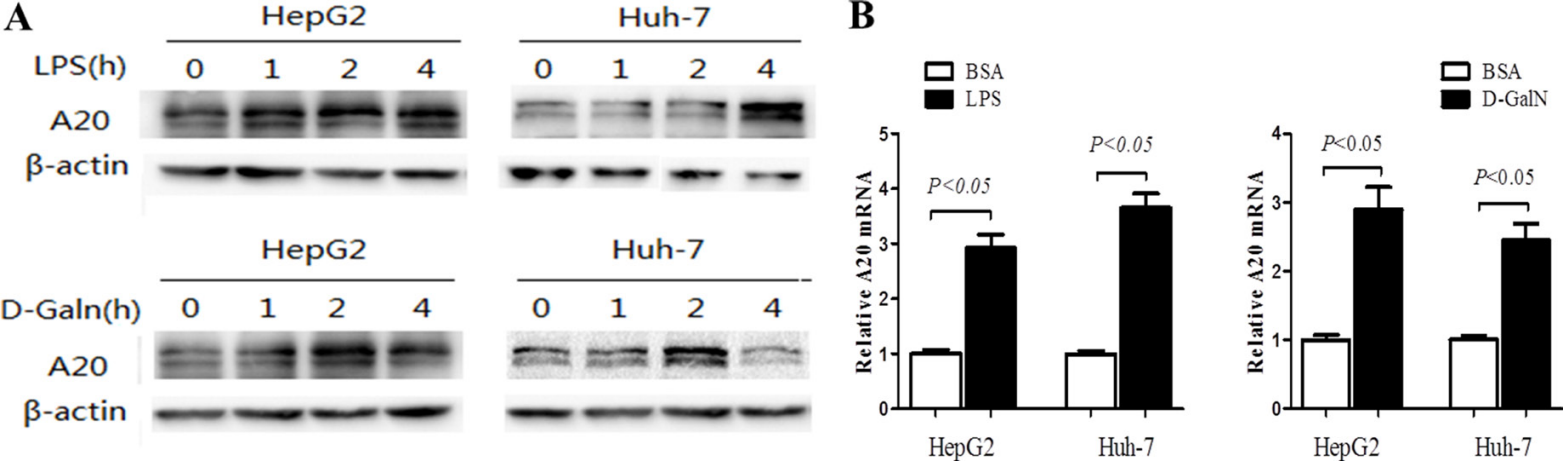
A20 expression is induced by LPS and D-GalN in hepatocytes (**A**) Dynamic change of A20 protein level was evaluated by immunoblot in HepG2 and Huh-7 cells treated with LPS (1 ug/mL), D-GalN (20 mmol/L) or 1% BSA as a control. (**B**) Relative A20 mRNA expression in HepG2 and Huh-7 cells treated with LPS (1 ug/mL), D-GalN (20 mmol/L) or 1% BSA for 24 hours was measured by real-time RT-PCR.

In order to study the role and the concrete mechanism of A20 in liver inflammatory injury, hepatocarcinoma cells HepG2 and Huh-7 were exposed to nonlethal dose of LPS (1 μg/mL) or D-GalN (20 mmol/L) for indicated time to induce inflammatory injury. Consistent with the up-regulation of A20 in hepatic injury *in vivo*, A20 expression rapidly increased in both HepG2 and Huh-7 cells after LPS or D-GalN stimulation (Figure 4A–4B).

### A20 affects the expression and secretion of inflammatory cytokines

**Figure 5:**
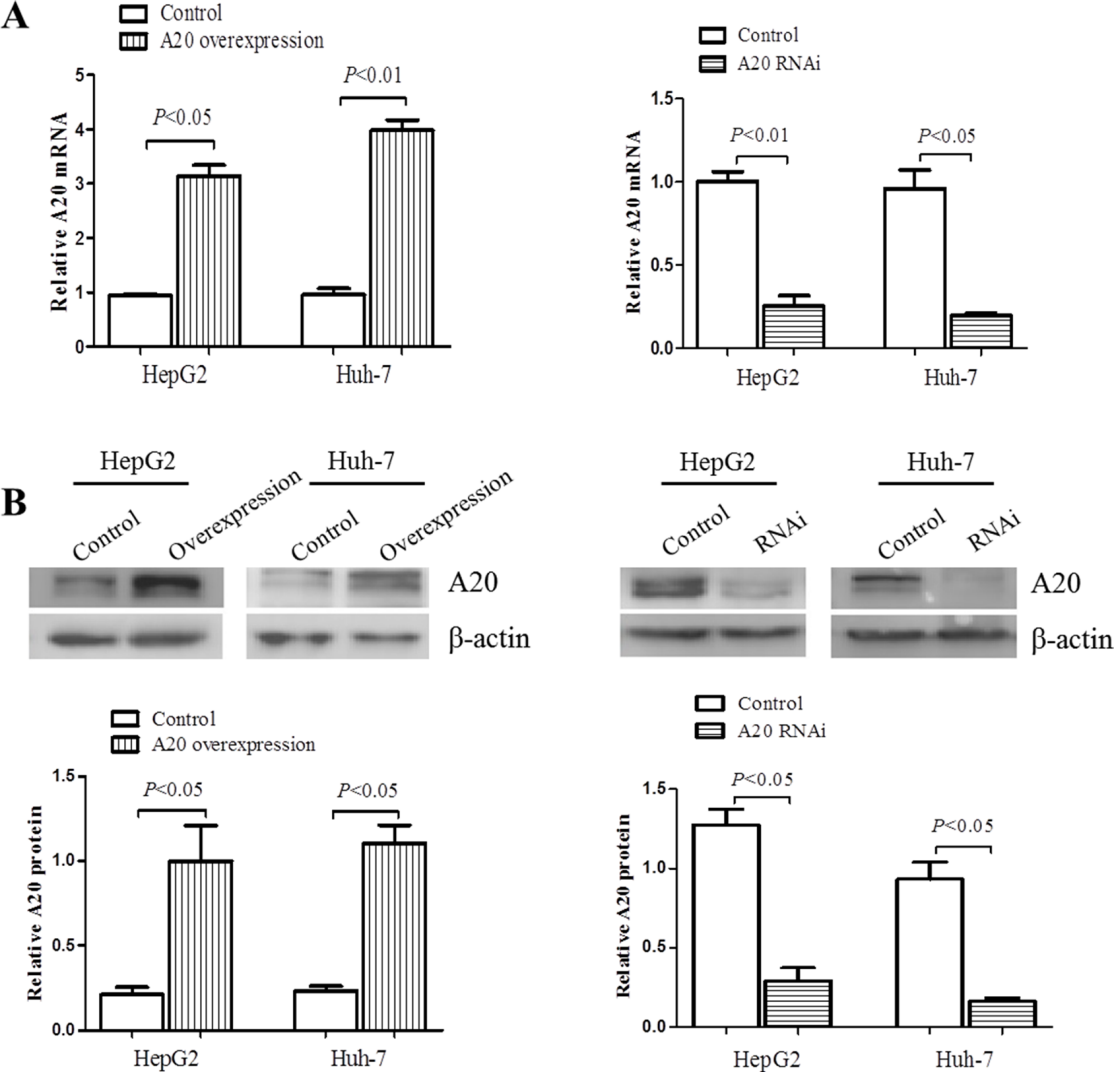
Overexpression or knockdown of A20 in hepatocarcinomo cells To overexpress or knock down A20 expression, the specific lentivirus (LV-A20 shRNA, LV-A20) was generated and harvested after 293T cells transfected by A20-RNAi lentiviral vector or human A20 vector and cultured. Then LV-A20 shRNA or LV-A20 was transfected into HepG2 and Huh-7 cells. After 72 h, A20 expression was determined through qRT-PCR and Western blot. Cells transfected by lentivirus with empty vector were used as scrambles. (**A**) Relative A20 mRNA level was measured by real-time PCR, using GAPDH as internal control. (**B**) Immunoblotting of A20 protein in HepG2 and Huh-7 cells. β-actin was determined as the loading control. Data are expressed as mean ± SD, *n* = 3 per group.

Previous studies have shown that A20 was distributed in several types of cells and suppress inflammation in a variety of diseases [23–26]. In order to study whether A20 protein could affect the inflammatory response in hepatic injury, we then generated stable cell lines with either overexpression or knockdown of A20 using HepG2 and Huh-7 cells transfected by lentivirus with constructed vectors. Figure 5A and 5B showed that A20 expression was higher by at least threefold in mRNA and 5 fold in protein level in cells transfected with LV-A20 than control, and was down-regulated more than 75% in mRNA and protein level in cells transfected with LV-A20 shRNA.

**Figure 6:**
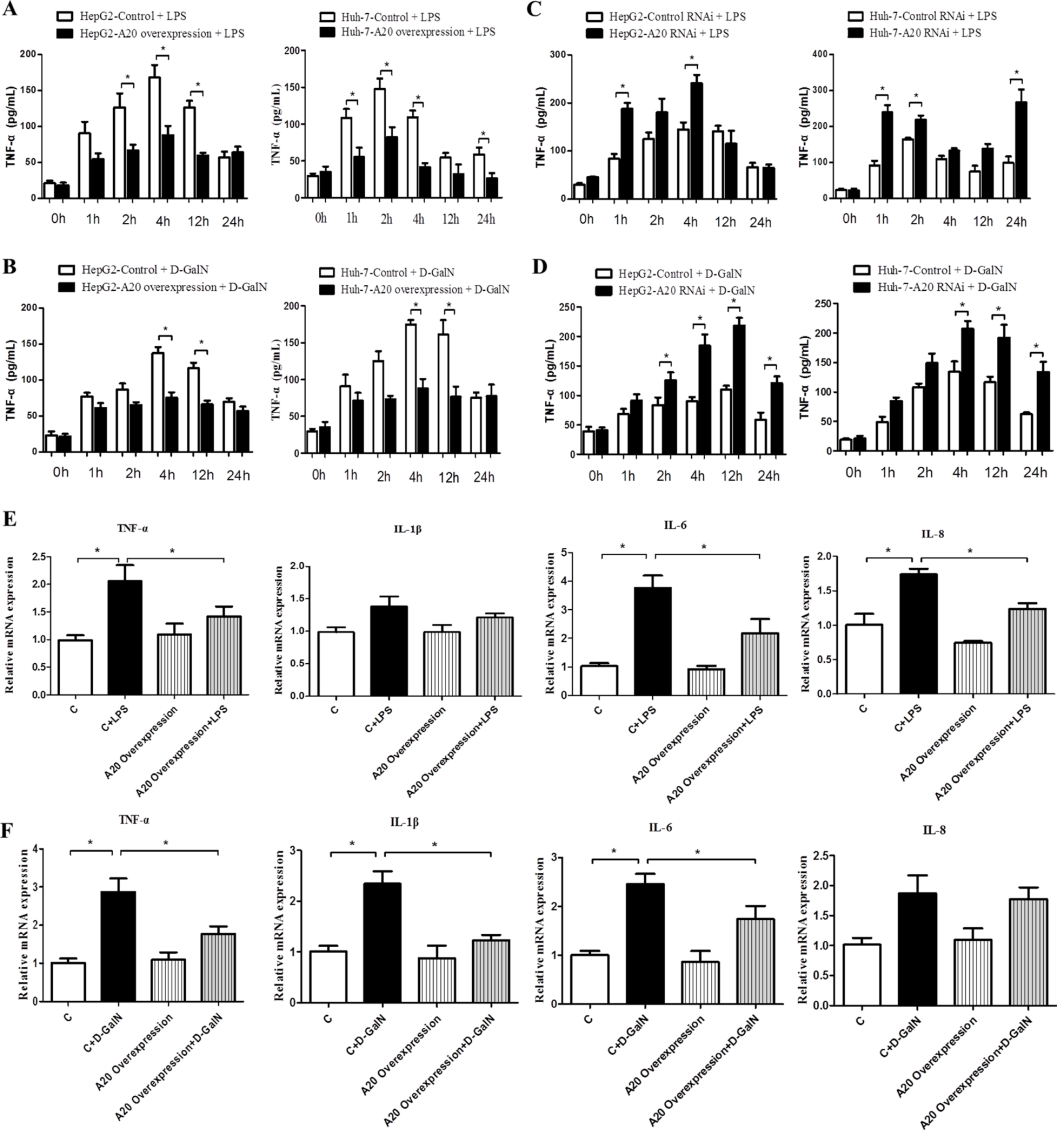
A20 affects the expression and secretion of inflammatory cytokines (**A**–**D**) Dynamic change of TNF-α level in the culture supernatant of HepG2 and Huh-7 cells with A20-overexpression or A20-knockdown after treatment with LPS (1ug/mL) or D-GalN (20 mmol/L), *n* = 3 per group. (**E**) Real-time PCR analysis of the expression of pro-inflammatory cytokines TNF-α, IL-1β, IL-6 and IL-8 in A20-overexpression HepG2 cells or controls after treatment of LPS or D-GalN for 24 hours, *n* = 3–4 per group. (**F**) Expression of pro-inflammatory cytokines in A20-overexpression Huh-7 cells or controls after treatment of LPS or D-GalN for 24 hours. Data are expressed as mean ± SD, *n* = 3–4 per group, *P < 0.05 *vs*. control.

We then measured the dynamic change of TNF-α secretion in the supernatant of cell lines with different A20 level after stimulating with 1 μg/mL LPS or 20 mmol/L D-GalN. TNF-α secretion increased after LPS or D-GalN treatment, with higher expression during 2 h-12 h. Overexpression of A20 inhibited TNF-α release, especially during 2 h-12 h (Figure 6A–6B), and knockdown of A20 increased TNF-α secretion in both cell lines from 1 h-24 h (Figure 6C–6D). Figure 6E and 6F shows the mRNA level of pro-inflammatory cytokines in HepG2 cells with overexpressed A20 or control cells stimulated by LPS or D-GalN. A20 overexpression down-regulated the mRNA levels of TNF-α, IL-1β, IL-6 and IL-8 induce by LPS. These results showed that A20 may have the anti-inflammatory function in response to liver damage.

### A20 negatively regulates the activation of NF-κB

**Figure 7:**
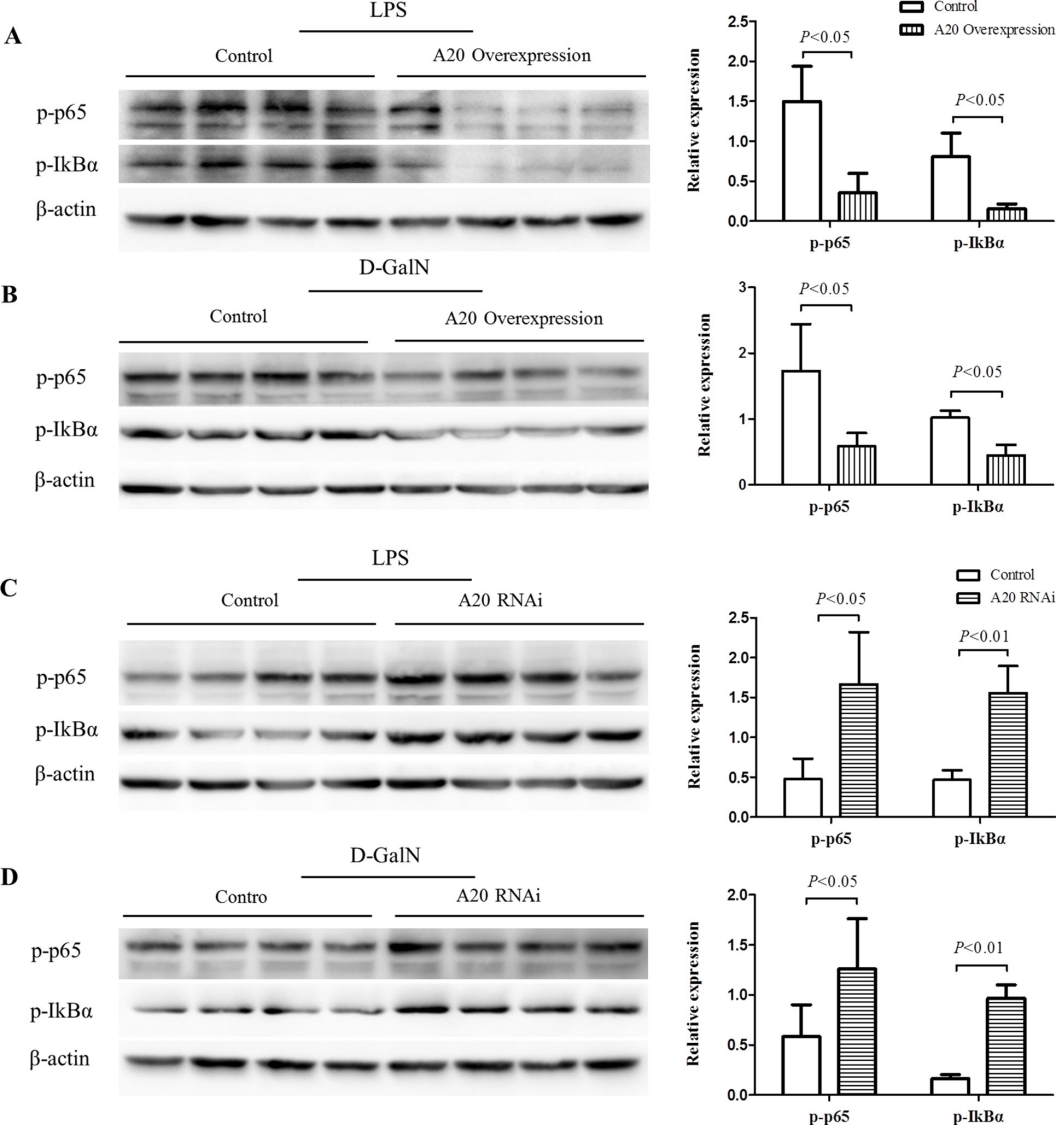
A20 negatively regulates the activation of NF-κB (**A**–**B**) Immunoblot analysis of p-p65, p-IκBα expression in HepG2-A20 overexpression cells and controls after treatment of LPS (1 ug/mL) or D-GalN (20 mmol/L) for 24 hours. (**C**–**D**) Immunoblot analysis of p-p65, p-IκBα expression in HepG2-A20-knockdown cells and controls after treatment of LPS (1 ug/mL) or D-GalN (20 mmol/L) for 24 hours. β-actin was used as loading control. Data are expressed as mean ± SD, *n* = 4 per group.

A20 was reported to inhibit the activity of NF-κB, which was crucial in the inflammatory response [27]. We thus analyzed the activation level of NF-κB signaling in HepG2 cells with A20 either knocked down or overexpressed. Induced by LPS or D-GalN, A20-overexpression cells demonstrated lower phospho-p65 and phospho-I-κBα levels compared with control (Figure 7A–7B). A20-knockdown cells enhanced NF-κB activation indicated by higher phospho-p65 and phospho-I-κBα levels (Figure 7C–7D). These results supported that A20 could inhibit the inflammatory response via regulating NF-κB activation in hepatic injury models *in vitro*.

## DISCUSSION

Chronic hepatitis B is characterized by the persistent virus replication and liver inflammation, which may lead to the disease progression to liver injury, fibrosis, cirrhosis and end-stage liver disease ultimately. In the present study, inflammatory score of liver sections from biopsies of CHB patients indicates the sustained inflammatory status in CHB. Previous researches have showed that the immune-inflammatory cytokines in the related liver damage of CHB correspond to a high-expression status, suggesting that pro-inflammatory cytokines play an important factor in the progression of disease [28, 29]. We also found significantly up-regulated serum TNF-α level in CHB patients compared with healthy controls. In addition, elevated serum activity of ALT, AST and γ-GT were determined in the patients with CHB exhibiting the liver injuries.

It is well-known that NF-κB contributes crucially to the detrimental inflammatory reactions in liver diseases. The NF-κB responsive protein A20 (or TNFAIP3), is essential for attenuating NF-κB signaling in response to many of the inflammatory mediators such as TNF-α, IL-1, LPS, muramyl dipeptide, etc. [20]. It could be induced with the stimulation of these inflammatory mediators which are kept at higher level during hepatitis and liver injury. A number of studies have shown that A20 was significantly up-regulated in peripheral blood mononuclear cells of CHB and the progressive acute-on-chronic hepatitis B liver failure (ACHBLF), liver cirrhosis and HCC patients, and in the liver tissues of patients with nonalcoholic steatohepatitis (NASH) [23, 30, 31].

In the present study, we found the obvious up-regulation of A20 level in serum of patients with CHB compared to the healthy controls. Moreover, its level was positively correlated with serum ALT, AST and inflammatory cytokine TNF-α in patients with CHB. It is higher in the patients with liver inflammatory grade G3-G4 compared with grade G1-G2. These data indicate that serum A20 is associated with the severity of CHB. This is similar with the results of Fan YC et al. In their study, A20 mRNA expression in peripheral blood mononuclear cells was found associated with dynamic progression of chronic HBV infection [21]. In the meantime, this study suggests that the elevated serum A20 might be partially due to the circulating inflammatory cells. Other origin must include the liver with lesions of inflammation and necrosis, which is confirmed by our immunohistochemistry results of A20 in liver biopsies from CHB patients. Both hepatocytes and inflammatory cells expressed more A20 during HBV infection. We also find serum A20 level is higher in CHB patients with HBeAg (−) than HBeAg (+). CHB with HBeAg (−) is usually considered with a worse outcome than HBeAg (+) [32]. Thus, this also support that the serum level of A20 might parallel with the severity of this disease. On the other hand, as a potential biomarker to determine the different stages of CHB, it will be more convenient to detect serum A20 directly than to isolate PBMC and amplify its mRNA. However, more CHB cases with different progressive stage are needed for further analysis to validate this serological biomarker for CHB progression.

A20 expression is also induced in our experiments *in vivo* and *in vitro* by D-GalN or LPS. As a disrupting chemical for uracil nucleoside phosphate, D-GalN could result in diffuse necrosis and inflammation of the liver, with the similar hepatic pathological changes of hepatitis caused by HBV. It had been reported that A20 was significantly up-regulated in mouse models of acute toxic hepatitis administrated by D-GalN or LPS [12]. Consistently, besides up-regulated pro-inflammatory cytokines expression and release in serum, our study showed elevated levels of the hepatic A20 expression as well as the secreted A20 in serum in D-GalN induced models. The serum ALT, AST and TNF-α level kept increased after D-GalN treatment, indicating the accumulation of liver damage. However, A20 rapidly increased and reached the peak at 2 h after D-GalN injection, and A20 protein in liver tissues increased to the peak value at 6 h after stimulation, but subsequently both decreased gradually. This might be owing to the short-term stimulation cannot induce sufficient A20 production and release. Moreover, A20 was also induced in two hepatocarcinoma cell lines by the inflammatory activators D-GalN or LPS. But the detailed mechanism of the dynamic change of A20 and the different function of this molecule produced in different cells need further study.

The goal of therapy for CHB infection is to improve quality of life and survival in infected persons by preventing disease progression to cirrhosis, end-stage liver disease, hepatocellular carcinoma (HCC) and death [1]. To gain this goal, in addition to target the viral life cycle, modulating host immune inflammatory response is one important approach. A20 is an identified negative regulatory molecule of the immune response. It might be a protective response of hepatocytes or immune cells to various toxic insults, which mediated hepato-protective function to maintain immune homeostasis. However, the NF-κB dependent induction of A20 is insufficient to anti-inflammation during CHB or other liver disease, more level of A20 might help to get better prognosis. Previous study has showed that the survival rate of mice suffering from acute toxic hepatitis was 15%-20%, while overexpression of A20 increased the survival rate to 85% [11, 12].

As a negative feedback regulator of NF-κB signaling [33], induced by NF-κB dependent signals, A20 in turn restricts the duration and intensity of the signaling by several molecules involved in NF-κB pathway [18]. NF-κB can be induced by a variety of stimulating factor and rapidly from inhibition into active state, thereby induce the expression of a variety of pro-inflammatory cytokines, such as TNF-α, IL-6 [34–36]. Recent studies reported that A20 overexpression promoted the release of FFAs-promoted pro-inflammatory cytokines from hepatocytes, such as IL-8, IL-1β, and TNF-α. These cytokines play critical roles in the development of hepatic inflammation [23]. In our results, A20 obviously influences the production of pro-inflammatory cytokines from HepG2 and Huh-7 cells after external stimulation. Secretion of TNF-α was suppressed by A20 overexpression but enhanced by A20 knockdown. Furthermore, overexpression of A20 significantly down-regulated the hepatic mRNA expression of TNF-α, IL-6 and IL-8 in HepG2 cells with both LPS and D-GalN treatment. This suggests A20 is a key factor to inhibit the production of pro-inflammatory cytokines in liver. Through combining with the NF-κB activation inhibitory factor to suppress the degradation of I-κBα, A20 inhibits the activation of NF-κB and its entrance into nuclei, thus suppresses NF-κB-mediated inflammatory responses [13, 37, 38]. This is supported by our study, in which both LPS- and D-GalN-induced activation of NF-κB could be impaired by A20 overexpression and increased by A20 knockdown. The regulatory effects of A20 to reduce the expression of pro-inflammatory cytokines in hepatocytes were probably via the regulation of NF-κB activation.

Notably, using A20 heterozygous mice, or mice that transiently overexpress an A20 cDNA, A20 in liver has been shown contributing to liver regeneration after partial hepatectomy [12, 39–41]. It also acts as a major cytoprotective protein for hepatocytes in inflammatory and cytotoxic conditions, protecting the cells from necrosis and apoptosis [42, 43]. A20 counteracts apoptosis via the modulation of caspase-8 ubiquitination and subsequent apoptotic signaling related molecules [20]. Therefore, although we pay more attention on its anti-inflammatory role in this study, A20 might anti hepatitis through combining the functions of anti-apoptosis and pro-proliferation. Further studies are needed to clarify the crisscrossed underlying mechanism.

In summary, our results reveal that the expression of A20 is up-regulated and serum A20 level is positively correlated with the severity of liver damage in CHB patients. This suggests that A20 may be involved in the pathogenesis of CHB, and might be a serological biomarker for the prognosis of CHB. Furthermore, acting as an essential anti-inflammatory mediator through inhibiting the NF-κB signaling activation and release of pro-inflammatory cytokines, A20 could attenuate the pathogenesis of liver damage. Thus, it may serve as a candidate therapeutic strategy for preventing the progress of CHB.

## MATERIALS AND METHODS

### Participants and samples

A total of 205 patients with CHB from Department of Hepatology, Foshan Hospital of Traditional Chinese Medicine, Guangzhou University of TCM between March 2012 and January 2014 were enrolled in this study. And 60 healthy adults who took annual health examinations in this hospital were used as healthy controls (HCs). Serum from each participant was collected and stored at −80°C until further analysis. Formalin-fixed paraffin-embedded tissue specimens of 205 CHB patients were from ultrasonically guided liver biopsy in order to make diagnosis for inflammatory stage of liver tissue. In addition, 5 paraffin-embedded relative normal liver tissue specimens from healthy individuals who underwent surgery or liver biopsy were used as control. CHB was defined as a positive hepatitis B surface antigen (HBsAg) for at least 6 months according to the 2009 update of the American Association for the Study of Liver Diseases (AASLD) Practice Guidelines for Chronic Hepatitis B. Informed consent was obtained from each participant and the study protocol approved by the Medical Ethical Committee of Longhua Hospital, Shanghai University of TCM.

### Murine model of liver injury

Male C57BL/6 mice weighing 18 g to 25 g were purchased from Shanghai SLAC Laboratory Animal Technology Company (License No. SCXK (HU) 2014–0005). The animals were housed in a standard 12 h light/dark cycle at 22 ± 2°C with 55 ± 10% humidity and had access to standard food and water. A total of 50 C57BL/6 mice were adaptively fed for 7 days and were randomly allocated into the five groups (*n* = 10 per group) according to their body weights, including the control group and four groups of hepatitis induced by intraperitoneally injection of D-GalN (800 mg/kg body weight). The mice were sacrificed at 2, 6, 12, 24 h after D-GalN injection respectively. Control mice were given the same volume of saline. The serum was separated, and liver tissues were frozen or fixed in 10% formalin for further investigation. The animal experiments were approved by the Institutional Animal Care and Use Committee of Shanghai University of Traditional Chinese Medicine. All animal procedures were performed in accordance to the guide for the care and use of laboratory animals [44].

### Serum biochemical analysis and A20, TNF-α determination

Serum activity levels of alanine aminotransferase (ALT), aspartate aminotransferase (AST) and γ- glutamyltransferase (γ-GT) were measured using HITACHI 7170S biochemistry analysis equipment. Hepatitis B e antigen (HBeAg) was measured by an automatic analyzer (Roche, Basel, Switzerland). The HBV DNA was determined with PCR System (Applied Biosystem, Foster City, USA). Serum concentration of A20 and TNF-α were measured by ELISA kit (West Tang biological technology, Shanghai, China) according to the manufacturer's instructions.

### Cell culture and stimulation with LPS or D-GalN

HepG2 and Huh-7 cell lines were purchased from the Cell Biology Institute of Chinese Academy of Science, Shanghai, China. HepG2 and Huh-7 cells were cultured in DMEM (Gibco BRL, Rockville, MD), supplemented with 10% fetal bovine serum, penicillin (100 U/mL), and streptomycin (100 μg/mL) (Gibco), at 37°C in a humid incubator with 5% CO_2_. Lipopolysaccharide (LPS) (Sigma Aldrich, St. Louis, MO, USA) at 1ug/mL final concentration or D-GalN (Sigma) used at 20mmol/L were used to simulate to induce cell injury. Cells and supernatant were harvested at different time point after stimulation.

### Knockdown and overexpression of A20 in hepatocarcinoma cells

In order to obtain stable cells with A20 knockdown or overexpression, three A20-RNAi lentiviral vectors and human A20 vector were constructed by GeneChem Co, Ltd (Shanghai, China) using the vector GV248 and GV287 respectively. Then the specific lentivirus (namely, LV-A20 shRNA and LV-A20, respectively) was generated and harvested after 293T cells were transfected by the vector and cultured for 48 h. To establish the stable cell line, the A20-RNAi lentivirus was transfected into HepG2 and Huh-7 cells with a multiplicity of infection (MOI) of 10. After 72 h, the transfection efficiency was observed through a fluorescence microscope, and A20 expression was determined through Western blot and qRT-PCR. Cells transfected by lentivirus with empty vector were used as controls. The inserted A20 sequence was from NM_006290. And the sequence of the most efficient shRNA targeting A20 was 5′- ACCGATACACACTGGAAAT-3′.

### RNA extraction and quantitative reverse transcription-polymerase chain reaction (qRT-PCR)

The tissue specimen ground in liquid nitrogen or the cultured cells were homogenized in Trizol (Invitrogen, Carlsbad, CA, USA). The total RNA was converted to cDNA by using reverse transcription kits (Promega, Madison, WI, USA). All primers were synthesized by Shanghai Shine Gene Company. The sequences of the primers used in this study are indicated in Table 2. Quantitative RT-PCR was then performed using a SYBR Green PCR Master Mix kit (Toyobo, Osak, JAPAN) according to the manufacturer's protocol. Amplification of β-actin was performed in parallel as a relatively invariant internal reference. The 2^−ΔΔCt^ method was applied for data analysis.

**Table 2:** The primer sequences for quantitative RT- PCR used in this study

Gene	Primer Sequence
mβ-actin	Forward: 5′-GAGACCTTCAACACCCCAGC-3′ Reverse: 5′-ATGTCACGCACGATTTCCC-3′
mTNF-α	Forward: 5′-CCCTCCAGAAAAGACACCATG-3′ Reverse: 5′-CACCCCGAAGTTCAGTAGACAG-3′
mIL-1β	Forward: 5′-TCGTGCTGTCGGACCCAT-3′ Reverse: 5′-GGCTTGTGCTCTGCTTGTGA-3′
mIL-6	Forward: 5′-GGGACTGATGCTGGTGACAAC-3′ Reverse: 5′-CAACTCTTTTCTCATTTCCACGA-3′
mIL-8	Forward: 5′-GGCATCTTCGTCCGTCCC-3′ Reverse: 5′-GCCAACAGTAGCCTTCACCC-3′
hGAPDH	Forward: 5′- ATCCCATCACCATCTTCCAGG-3′ Reverse: 5′- GATGACCCTTTTGGCTCCC-3′
hA20	Forward: 5′- AATGCTAAGAAGTTTGGAATCAGG-3′ Reverse: 5′- CCGAGAACAATGGGGTATCTG-3′
hTNF-α	Forward: 5′- CACGCTCTTCTGCCTGCTG-3′ Reverse: 5′- GGCTTGTCACTCGGGGTTC-3′
hIL-1β	Forward: 5′-AAATGATGGCTTATTACAGTGGC-3′ Reverse: 5′-CTTGCTGTAGTGGTGGTCGG-3′
hIL-6	Forward: 5′- CACTGGTCTTTTGGAGTTTGAGG-3′ Reverse: 5′- TGGGTCAGGGGTGGTTATTG-3′
hIL-8	Forward: 5′- GACATACTCCAAACCTTTCCACC-3′ Reverse: 5′- AACTTCTCCACAACCCTCTGC-3′

### Western blot analysis

Liver tissues or cells were homogenized in RIPA buffer with the proteinase inhibitor (Complet mini EASY pack, Roche, Basel, Swiss) and protein concentration was determined through BCA method (CoWin Bioscience, Beijing, China). The lysates were resolved on 12% sodium dodecyl sulfate–polyacrylamide by gel electrophoresis (SDS–PAGE) and transferred to a PVDF membrane (Millipore, Billerica, MA, USA). After blocking, membranes were immunobloted with the antibody against A20 (Abcam, Cambridge, UK, ab13597), Phospho-NF-kB p65 (Ser536) (Cell signaling technology, Boston, MA, USA, 3033), Phospho-IkBα (Ser32) (CST, 2859) or β-actin (Huaan biological technology, Hangzhou, China) as internal loading control. The primary antibodies were visualized with goat anti-rabbit (CST) peroxidase-conjugated antibody by an enhanced chemiluminescence detection system (Millipore, Billerica, MA, USA). The images of blots were acquired by GBOX Chemi XT4 System (Syngene, Cambridge, UK), and quantified by densitometry with GeneTools software (Syngene).

### Histological examination

The liver sections were stained with hematoxylin–eosin (HE) according to the standard methods. In brief, the fresh liver tissue samples were fixed in 10% formalin and embedded in paraffin. The samples were cross-cut into slices of 4 μm to 5μm and stained with HE staining solution. Finally, the stained sections were observed and photographed under a light microscope (with 200× magnification). HE stained liver sections of CHB patients were evaluated in a blinded manner by two pathologists to quantify the degree of inflammation according to the METAVIR System Algorithm for Evaluation of Histological Activity.

### Immunohistochemistry

Expression of A20 was determined by immunohistochemistry using a primary antibody against A20 (Abcam, Cambridge, UK, ab13597). After deparaffination and rehydration, the sections were subjected to antigen retrieval (10 mM Citric Acid Buffer, pH 6.0). The endogenous peroxidase activity was blocked using 30% H_2_O_2_. Then the sections were incubated with primary antibody in 2.5% BSA (Sigma) at 4°C overnight. The secondary antibody (Dako Deutschland GmbH, Hamburg) was applied for 30 min at room temperature. Visualization of the signal was performed using the DAB-Substrate-Kit (Dako). Slides were counter stained with hematoxylin (Yixin Biological Technology, Shanghai, China). The number of A20 positive cells per 200× field was counted using ImageJ automated or manual cell counting.

### Statistical analysis

The data were expressed as mean ± standard deviation (SD). Statistical analyses were carried out using one-way ANOVA, followed by Tukey's post-hoc test to assess the differences between the two groups. The correlation of the associations between the expression of A20 and various parameters was calculated according to Spearman. The data from qPCR, Western blot and pathological scores were compared using Kruskal-Wallis ANOVA test followed by Dunn's multiple comparison test. SPSS 18.0 was used for all statistical analyses. *P* < 0.05 was considered statistically significant.
